# Editorial: Research on precision prevention and treatment of neurological and neurodegenerative diseases

**DOI:** 10.3389/fnins.2026.1975792

**Published:** 2026-09-17

**Authors:** Lei-Yun Wang, Chunrun Qu

**Affiliations:** 1 Department of Pharmacy, Traditional Chinese and Western Medicine Hospital of Wuhan, Tongji Medical College, Huazhong University of Science and Technology, Wuhan, Hubei, China; 2 Department of Pharmacy, Wuhan No.1 Hospital, Wuhan, Hubei, China; 3 Ludwig Institute for Cancer Research, Nuffield Department of Medicine, University of Oxford, Oxford, United Kingdom

**Keywords:** causal relationship, genetics, neurological and neurodegenerative diseases, prevention, treatment

The development and progression of neurological and neurodegenerative diseases involve multiple complex pathophysiological processes, including autoimmunity, inflammation, and various forms of cell death such as ferroptosis. Distinct preventive and therapeutic strategies, including traditional Chinese medicine, have been developed based on different pathogenic mechanisms. To promote more individualized prevention and treatment of neurological and neurodegenerative diseases, the studies in this Research Topic aimed to identify and analyze novel risk factors, elucidate the pathogenic mechanisms, and pinpoint drug targets. The contributing articles are briefly introduced below.

Zhang Q. et al. identified STAT3 signaling and an associated inflammatory signature as potential therapeutic targets in Dravet syndrome (DS), a severe developmental and epileptic encephalopathy. Transcriptomic surrogates of the systemic inflammatory indices neutrophil-to-lymphocyte ratio (NLR) and systemic immune-inflammation index (SII) constructed from myeloid/neutrophil-like activation (*S100a9, Fcgr3, Csf3r*), lymphoid activation (*Lck, Zap70*), and platelet-related (*Itga2b, Gng11*) gene signatures, were evaluated alongside serological inflammatory markers in a pediatric cohort, in which C-reactive protein (CRP) was independently associated with drug resistance. *In vivo* validation confirmed p-STAT3 hyperactivation in hippocampal gliosis of *Scn1a*+/– mice. These findings highlight the potential of adjunctive anti-inflammatory therapies for DS.

Du et al. investigated predictors of lacosamide (LCM) efficacy in Chinese children with epilepsy, focusing on the concentration-to-dose ratio (CDR), calculated as the ratio of serum LCM concentration to the weight-adjusted daily dose. The effective group showed a significantly higher CDR than the ineffective group, and ridge regression analyses incorporating daily dose, serum concentration, concomitant antiseizure medications, age, and weight yielded acceptable predictive performance (area under the curve = 0.767, 95% confidence interval: 0.651–0.875). These findings support CDR-guided individualized LCM therapy in pediatric populations.

Ding et al. demonstrated that Tuina, a traditional Chinese manual therapy aimed at restoring physiological balance, effectively ameliorates sleep disturbances. Daily Tuina stimulation of Governor Vessel (GV) acupoints (GV20 [Baihui], GV14 [Dazhui], GV6 [Jizhong]) and bilateral Bladder Meridian (BL) acupoints (BL15 [Xinshu], BL17 [Geshu], BL20 [Pishu], BL23 [Shenshu]) ameliorated p-chlorophenylalanine (PCPA)-induced sleep disturbances in rats and protected against hippocampal neuronal damage. Mechanistically, Tuina suppressed PCPA-induced Piezo1 upregulation and downstream calcium signaling, and these benefits were reversed by the Piezo1 agonist Yoda1, indicating that Tuina acts through Piezo1-mediated calcium signaling. This study provides *in vivo* evidence that traditional Tuina therapy engages defined neurological pathways.

Chen et al. showed that Cang-ai volatile oil (CAVO), a compound traditional Chinese medicine composed of Cangzhu, Ai Ye, Huo Xiang, Pei Lan, Clove, and other ingredients, ameliorates vascular cognitive impairment (VCI). In a rat VCI model induced by permanent bilateral common carotid artery ligation (two-vessel occlusion, 2VO), CAVO was compared with donepezil as a positive control. Similar to donepezil, CAVO improved performance in the Morris water maze, reduced hippocampal expression of the inflammasome marker NLRP3, pro-apoptotic BAX, and endoplasmic reticulum stress-related proteins (CHOP, PERK, GRP78) while restoring Bcl-2, ameliorated hippocampal histopathology, and enhanced regional functional connectivity detected by 7T resting-state functional magnetic resonance imaging. These findings suggest that traditional Chinese medicines such as CAVO warrant further attention regarding their therapeutic effects and potential mechanisms in neurological and neurodegenerative diseases.

A unifying mechanistic theme of this Research Topic is that neurological dysfunction is rarely confined to neurons alone, but emerges from the interplay of systemic inflammation, vascular compromise, and metabolic disturbance. This theme is already evident in the studies highlighted above, from the peripheral inflammatory signature accompanying STAT3 activation in Dravet syndrome to the cerebrovascular injury underlying vascular cognitive impairment, and is further developed by the following contributions. Zou et al. integrate evidence from pulmonary medicine, coagulation biology, and stroke pathology into a lung-to-brain immunothrombosis framework in which thromboinflammation, neutrophil extracellular traps, and endotheliopathy couple peripheral disease to stroke and neurodegeneration; in parallel, Liu et al. delineate how iron overload, lipid peroxidation, and amino acid dysregulation drive ferroptosis in diabetes-associated cognitive dysfunction, while Bossardet et al. provide experimental evidence that collagen mimetic peptides preserve cerebrovascular and blood-brain barrier integrity in diabetic atherosclerosis. Metabolic control of brain function is further examined by Biswas and Iffland, who review disrupted macromolecule processing and mitochondrial metabolism in mTOR pathway-associated cortical malformations, and by Zhou and Wang, who discuss glutamate dehydrogenase as a link between glutamate homeostasis and brain aging. The gut-brain axis emerges as a shared therapeutic interface: Li X. et al. organize pharmacological strategies targeting the microbiota-gut-brain axis according to their primary mechanisms, whereas Qin et al. and Li G. et al. converge on tryptophan metabolism and its kynurenine branch, examined in the contexts of ischemic stroke and of exercise-based prevention and treatment of Alzheimer's disease, respectively, illustrating how a single metabolic pathway may be leveraged across distinct indications. At the receptor and clearance levels, Jiang et al. dissect nicotinic acetylcholine receptor subtypes underlying nicotine addiction, and Zhang S.-S. et al. discuss how dexmedetomidine may enhance glymphatic clearance to prevent postoperative cognitive dysfunction. Together, these contributions outline a mechanistic continuum from peripheral triggers to central vulnerability that may help prioritize druggable nodes for precision intervention.

A second theme is the re-examination of traditional and natural-product therapies through modern mechanistic lenses, extending the approaches exemplified by Ding et al. and Chen et al. above. Lu Y. et al. review how natural antidepressants act through epigenetic modifications, including DNA methylation, histone modification, and non-coding RNAs in major depressive disorder, and Gao et al. summarize the pathogenesis of post-stroke depression together with the multi-target mechanisms of natural product-based interventions. Zhang Y. et al. review the antioxidant, anti-inflammatory, and neuroprotective mechanisms of dihydromyricetin across neurological disorders. Complementing these reviews with experimental screening, Ucar Akyurek et al. evaluated 37 plant phenolics and identified 3-hydroxytyrosol as a cholinesterase inhibitor with *in vivo* antiamnesic activity, while Dahalia et al. synthesized physostigmine analogs that modulate Klotho and downstream STAT3/BAX/Bcl-2 signaling, conferring anticonvulsant and neuroprotective effects in pentylenetetrazol (PTZ)-kindled mice. Collectively, these studies illustrate a shift from empirical use toward mechanism-defined, multi-target application of natural products, although standardized quality control and clinical validation remain essential next steps.

Finally, several studies embody precision medicine in practice, spanning biomarker-guided therapy, pharmacogenetics, real-world evidence, and experimental models. In pediatric epilepsy, the biomarker strategies of Du et al. and Zhang Q. et al. are complemented by Lin et al., who show that serum insulin-like growth factor-1 (IGF-1), insulin-like growth factor-binding protein-3 (IGFBP-3), and their ratio correlate with short-term adrenocorticotropic hormone response in infantile epileptic spasms syndrome. Zampatti et al. discuss how APOE genotyping-used to estimate the risk of amyloid-related imaging abnormalities under anti-amyloid monoclonal antibodies-blurs pharmacogenetics with germline predictive testing and calls for dedicated genetic counseling. Real-world data provide complementary evidence: Yu et al. characterize neurological adverse events of 33 antidepressants across seven classes in a 22-year analysis of the FDA Adverse Event Reporting System (FAERS), revealing class-specific signals and onset-time patterns, and Hao et al. identify a first-day mean intracranial pressure of 14 mmHg as a cut-off associated with intensive care unit mortality in subarachnoid hemorrhage using the MIMIC-IV database. At the level of individualized care, Lu J. et al. report a pharmacist-led precision framework for managing invasive fungal infection after allogeneic hematopoietic stem cell transplantation in CSF1R-related leukoencephalopathy. Looking ahead, Zheng et al. review how induced pluripotent stem cell (iPSC)-derived brain organoids integrated with CRISPR-Cas9 and multi-omics support individualized drug-response prediction, and Guo et al. map two decades of global research on amyotrophic lateral sclerosis biomarkers, therapeutic targets, and drugs, highlighting priorities such as neurofilament light chain and antisense oligonucleotide therapies. Together, these contributions bridge bedside decision-making, population-level evidence, and preclinical modeling, providing references for elucidating the underlying mechanisms of neurological and neurodegenerative diseases and for developing new possibilities for precision medicine.

In summary, researches on neurological and neurodegenerative diseases continues to advance, with new mechanisms, pathways, biomarkers, and therapeutic approaches constantly being revealed. As a Research Topic dedicated to precision prevention and treatment of these diseases, this Research Topic provides new focal points for precision medicine, and we hope that readers will find valuable insights within this Research Topic.

